# Mitochondrial hypermetabolism precedes impaired autophagy and synaptic disorganization in *App* knock-in Alzheimer mouse models

**DOI:** 10.1038/s41380-023-02289-4

**Published:** 2023-11-01

**Authors:** Luana Naia, Makoto Shimozawa, Erika Bereczki, Xidan Li, Jianping Liu, Richeng Jiang, Romain Giraud, Nuno Santos Leal, Catarina Moreira Pinho, Erik Berger, Victoria Lim Falk, Giacomo Dentoni, Maria Ankarcrona, Per Nilsson

**Affiliations:** 1 Department of Neurobiology, Care Sciences and Society, Division of Neurogeriatrics, Center for Alzheimer Research, Karolinska Institutet, Stockholm, Sweden; 2 Centre for Translational Microbiome Research and National Pandemic Center, Department of Microbiology Tumor and Cell Biology, Karolinska Institutet, Stockholm, Sweden; 3 Department of Laboratory Medicine, Karolinska Institutet, Huddinge, Sweden; 4 Department of Medicine, Karolinska Institutet, Huddinge, Sweden; 5 Department of Otolaryngology Head and Neck Surgery, The First Hospital of Jilin University, Changchun, China; 6 Present Address: Department of Neurology, Inselspital, Bern University Hospital, University of Bern, Bern, Switzerland

**Keywords:** Neuroscience, Molecular biology

## Abstract

Accumulation of amyloid β-peptide (Aβ) is a driver of Alzheimer’s disease (AD). Amyloid precursor protein (*App*) knock-in mouse models recapitulate AD-associated Aβ pathology, allowing elucidation of downstream effects of Aβ accumulation and their temporal appearance upon disease progression. Here we have investigated the sequential onset of AD-like pathologies in *App*^*NL-F*^ and *App*^*NL-G-F*^ knock-in mice by time-course transcriptome analysis of hippocampus, a region severely affected in AD. Strikingly, energy metabolism emerged as one of the most significantly altered pathways already at an early stage of pathology. Functional experiments in isolated mitochondria from hippocampus of both *App*^*NL-F*^ and *App*^*NL-G-F*^ mice confirmed an upregulation of oxidative phosphorylation driven by the activity of mitochondrial complexes I, IV and V, associated with higher susceptibility to oxidative damage and Ca^2+^-overload. Upon increasing pathologies, the brain shifts to a state of hypometabolism with reduced abundancy of mitochondria in presynaptic terminals. These late-stage mice also displayed enlarged presynaptic areas associated with abnormal accumulation of synaptic vesicles and autophagosomes, the latter ultimately leading to local autophagy impairment in the synapses. In summary, we report that Aβ-induced pathways in *App* knock-in mouse models recapitulate key pathologies observed in AD brain, and our data herein adds a comprehensive understanding of the pathologies including dysregulated metabolism and synapses and their timewise appearance to find new therapeutic approaches for AD.

## Introduction

Alzheimer’s disease (AD) is the major form of dementia and leads to memory and cognitive deterioration. Brains from AD patients are characterized by disturbed protein homeostasis including extracellular deposits of amyloid β-peptide (Aβ) forming plaques, intracellular accumulation of hyperphosphorylated tau into neurofibrillary tangles [1, 2], and extensive neuroinflammation. These impairments culminate in synaptic loss and neurodegeneration which directly correlates with onset of the clinical symptoms. Recent Aβ immunotherapies have emerged as disease-modifying treatments, thereby strengthening the Aβ hypothesis, and they have received accelerated approvals. Profound reductions in fibrillar amyloid plaque pathology were achieved [3, 4], however moderate effects on cognition and function indicate that a multifaceted approach is likely necessary to achieve substantial clinical benefits. These data, together with more than 400 failed clinical trials, clearly show that the underlying disease mechanisms are yet to be fully understood [5].

Recent multi-omics studies of AD brain and biofluids gathered integrative efforts to understand upstream pathomechanisms which pointed out to a multi-level failure of networks. That includes but is not limited to inflammatory and immune response, perturbed synaptic plasticity and vesicle-mediated transport, as well as dysregulated lipid homeostasis and hypometabolism [6–8]. Despite the tremendous progress on the understanding of these biological signatures, studies conducted at clinical stage where the disease may have progressed silently over the course of decades likely mask early AD-driving mechanisms. We and others have hypothesized that mitochondrial alterations may occur in the prodromal phase of AD [9–11], leading to bioenergetic maladaptation and consequent neuronal damage.

Here we used *App*^*NL-F*^ and *App*^*NL-G-F*^ knock-in mice to perform a comprehensive longitudinal study of the hippocampus, a major area affected in AD. The *App* knock-in mice exhibit high levels of Aβ42 due to the Swedish (KM670/671NL) and Beyreuther (I716F) [12, 13] mutations inserted in the mouse *App* gene which specifically lead to generation of AD-causing Aβ42 species, whereas APP levels are unaltered thereby circumventing potential artefacts caused by APP overexpression paradigms applied in APP transgenic mice [14, 15]. Aβ plaque pathology starts from nine months-of-age in the *App*^*NL-F*^ mice, leading to synaptic loss around the Aβ plaques, and memory impairment at 18 months-of-age. To accelerate the Aβ oligomerization and its downstream effects, the Arctic (E693G) mutation was additionally inserted to generate the *App*^*NL-G-F*^ mice, which show earlier Aβ hippocampal deposition starting from two months-of-age, memory impairment from six months-of-age, and a more pronounced neuroinflammation. By combining transcriptomics, functional and imaging data, we here identified an early hypermetabolic phase in both mouse models occuring before the establishment of Aβ pathology. This was characterized by upregulated oxidative phosphorylation (OxPHOS) activity, followed by a homeostatic phase mainly observed in slow-progressing *App*^*NL-F*^ mice. After a robust Aβ pathology was established, a strong neuroinflammatory response, declining mitochondrial function, impaired autophagy, and ultimately synaptic disturbances were observed.

## Materials and methods

### Animals and ethical permits

Homozygous *App*^*NL-F/NL-F*^ (denoted herein *App*^*NL-F*^) and *App*^*NL-G-F/NL-G-F*^ (denoted herein *App*^*NL-G-F*^) [14] and *App*^*wt/wt*^ (WT) (all on C57BL/6 J background) were housed at Karolinska Institutet animal facility under conditions of controlled temperature (22–23 °C) and a 12-h light/12-h dark cycle. Food and water were available *ad libitum*. All experimental procedures were carried out in accordance with the guidelines of the Institutional Animal Care and Use of Committee and the European Community directive (2010/63/EU) and approved by Linköping Animal Ethical Committee (ID 407) and Stockholm Animal Ethical Committee (15758-2019). For this study two-, six-, 10- to 12-, 15- to 18- and 22- to 24-month-old females. Number of animals used for each experiment is described in the respective method section and figure legends.

### RNA isolation and sequencing

RNA was extracted from dissected hippocampal brain tissue (*n* = 3 females/genotype and age) preserved in RNAlater (AM7020, Thermo Scientific) using RNeasy Lipid Tissue Mini Kit (74804, Qiagen) according to manufacturer’s instructions. RNA quality (RNA integrity number, RIN) and quantity was measured in a Bioanalyzer 2100 (Agilent) using the Agilent RNA 6000 Nano Kit (part number 5067-1511). NEBNext Ultra II Directional RNA Library Prep Kit for Illumina (E7760S, New England Biolabs) was used to prepare the sequencing libraries, starting with 200 ng of total RNA. In short, mRNA was isolated and fragmented using the NEBNext poly(A) mRNA magnetic isolation module (E7490S, New England Biolabs). The first and second strands of cDNA were synthesized and purified using AmPure XP beads (A63880, Beckman Coulter). Adaptor ligation and size selection were performed according to the manufacturer’s protocol. Adaptor ligated cDNA was PCR enriched to incorporate an Illumina compatible index sequence (NEBNext Multiplex Oligos for Illumina, Dual Index Primers Set1, E7600S, New England Biolabs). The libraries were purified using AmPure XP beads, the size distribution of the libraries was analyzed with the Bioanalyzer 2100 using the Agilent High Sensitivity DNA Kit (part number 5067-4626). Quantification of the libraries was performed with the Qubit® 2.0 Fluorometer (ThermoFisher Scientific) and Qubit^TM^ dsDNA HS Assay Kits (Q32851, Invitrogen). Finally, all 30 libraries were pooled and diluted to 3.5 nM for sequencing on one lane of a Hiseq 3000 sequencer (Illumina), using a single read 50 bp and dual indexed sequencing strategy. On average the reads were 41.7 M to 49 M per sample.

### Sequence processing and differential gene expression analysis

All raw sequence reads available in FastQ format were mapped to the mouse genome (mm10) using Tophat2 with Bowtie2 option [16, 17], where adaptor sequences were removed using trim galore before read mapping. BAM files containing the alignment results were sorted according to the mapping positions. Raw read counts for each gene were calculated using featureCounts from Subread package [18]. DEseq2 was used to perform the analysis of differential gene expression, where genes with raw counts were used as input [19]. The differentially expressed genes (DEGs) were identified by adjusted *p-*value for multiple testing using Benjamini-Hochberg correction with False Discovery Rate (FDR) values less than 0.1.

### Gene ontology enrichment analysis

The significantly DEGs were applied to Gene ontology (GO) enrichment analysis using online software AmiGO website (http://amigo.geneontology.org/amigo), and the significant enrichment GO terms was identified using Fisher’s Exact test with Bonferroni corrected *p*-values ≤ 0.05. Identification of genes involved in mitochondrial function, neuroinflammation and autophagy was performed by using free online databases and software including Uniprot (http://uniprot.org), DAVID v6.8 (https://david-d.ncifcrf.gov/) and PANTHER v13.1 (www.pantherdb.org). Genes included within the GO terms (in all ontologies: biological processes, molecular function, cellular component) containing the word “autophag” were considered genes involved in the autophagosomal-lysosomal system, those containing the word “inflamm” were considered inflammation-related genes, those containing the word “mitochondri” were considered to be genes related to mitochondrial processes and used for further enrichment analysis.

### Pathway analysis

Gene Set Enrichment Analysis (GSEA) [20] was applied to perform pathway analysis using the KEGG pathways dataset. First, genes were ranked decreasingly according to the Log_2_ Fold Change (Log2FC) of expression. For each query pathway, if gene *i* is a member of the pathway, it is defined as

$${X}_{i}\, = \, -\sqrt{\frac{G}{N-G}}$$

If gene *i* is not a member of the pathway, it is defined as

$${X}_{i}\, = \, \sqrt{\frac{N-G}{G}}$$

where *N* indicates the total number of genes and *G* indicates the number of genes in the query pathway. Next, a max running sum across all *N* genes Maximum Estimate Score (MES) is calculated as

$${MES}=\mathop{\max }\limits_{1\le j\le N}\mathop{\sum }\limits_{i=1}^{j}{X}_{i}$$

The permutation test was performed 1000 times to judge the significance of MES values, where the null hypothesis is that the pathway is not enriched in ranking. If the query pathway with a nominal *p*-value less than 0.05 and adjusted *p*-value for multiple testing using Benjamini-Hochberg correction with FDR values less than 0.1, the null hypothesis would be rejected, and the query pathway would be considered as significantly enriched. MES value represents the expression direction of a pathway, where a positive MES value indicates up-enrichment (up-regulation) whereas a negative MES value indicates down-enrichment (down-regulation) of a pathway.

### Unsupervised genome-wide clustering

The raw read matrix distributed column-wisely with samples names and row-wisely with gene names, was normalized using between-samples normalization method implemented in DESeq2 package [19]. Unsupervised genome-wide clustering was then performed using *t*-SNE plot with the R package Rtsne [21]. The *t-*SNE plot is based on the 50 most variant dimensions of the initial PCA plot, wherein genes with duplicate reads are filtered out. The Speed/accuracy trade-off was set as 0.0 to exact *t-*SNE distance matrix. The perplexity is adjusted accordingly for the optimal clusters shape. Plots showing all samples are based on the *t*-SNE field parameters V1 and V2.

### cDNA reverse transcription (RT) and qPCR

100 ng of RNA was reverse transcribed using the High-Capacity cDNA Reverse Transcription Kit (Thermo Fisher Sci., Cat. no: 4374966) according to manufacturer’s instructions. The TaqMan Fast Advanced Master Mix (Thermo Fisher Sci., Cat. no: 4444557) was used to perform the qPCR using the following TaqMan mouse gene expression assays (FAM) (Thermo Fisher Sci., Cat. no: 4331182): Mm04225236_g1 for *Atp8*; Mm00432648_m1 for *Cox8b*; Mm00444593_m1 for *Ndufa1*; Mm01352366_m1 for *Sdha*; Mm01615741_gH for *Uqcrb*; Mm01183349_m1 for *Clec7a*; Mm00441259_g1 for *Ccl3*; Mm04209424_g1 for *Trem2*; Mm00437893_g1 for *C4b*; Mm01312230_m1 for *Chrna4*; Mm00495267_m1 for *Lamp2*; Mm00523599_g1 for *Rab7b*; Mm01310727 for *Rubcnl*; Mm01274264_m1 for *Trim30a*; Mn00450314_m1 for *Vamp8*. Experiments were performed in the 7500 Fast Real-Time PCR System (Applied Biosystems) and each sample was run in triplicates. Gene expression was normalized to *β3-tubulin* mRNA (Mm00727586_s1).

### Brain homogenates

Right hippocampi from the same individuals used for RNA-seq experiments (*n* = 3/genotype and age) were cut in pieces while kept cold on ice and homogenized with a 2 cm^3^ glass-Teflon homogenizer in RIPA buffer (150 mM NaCl, 50 mM Tris, 1% Triton X-100, 0.5% DOC, 0.1% SDS; pH = 7.5) supplemented with 1:100 protease inhibitors (G-Biosciences, Cat. no: 786-433) and 1:100 phosphatase inhibitors (Sigma–Aldrich, Cat. no: P0044) in a proportion of 1:15 (mg tissue/µl buffer). The homogenates were then sonicated for 15 s on ice and centrifuged at 20,800 *g* for 30 min, at 4 °C, to remove cell debris. Supernatant was collected and protein concentration determined by Pierce™ BCA Protein Assay Kit (Thermo Fisher Sci., Cat. no: 23225).

### Crude synaptosomal fractions

Crude synaptosomal fractions were isolated from dissected left hippocampal brain tissues (*n* = 4/genotype and age) by homogenizing the tissue with a 2 cm^3^ glass-Teflon homogenizer at 800 rpm in lysis buffer (0.32 M sucrose, 5 mM Hepes, and 10 ml ddH_2_O) supplemented with 1:100 diluted protease inhibitors (G-Biosciences, Cat. no: 786-433) and 1:100 diluted phosphatase inhibitors (Sigma–Aldrich, Cat. no: P0044) in a proportion of 1:10 (mg tissue/µl buffer). The homogenates were centrifuged at 1000 *g* at 4 °C for 10 min to remove nuclei and debris, and the supernatant was centrifuged again at 12,000 *g* at 4 °C for 20 min. Supernatant composed of the light membrane fraction and soluble proteins (S2) was transferred to a new tube, and the pellet containing crude synaptosomes and mitochondria (P2) was resuspended in 75 μl RIPA buffer supplemented with 1:100 diluted protease inhibitors and 1:100 diluted phosphatase inhibitors. The protein concentration was determined by Pierce™ BCA Protein Assay Kit (Thermo Fisher Sci., Cat. no: 23225).

### Western blotting

15 to 20 µg of protein were loaded and separated by 4–12% Bis-Tris gels (Novex, Cat. no: NP0336BOX) or 4–20% Tris-Glycine extended gels (BIO-RAD, Cat. no: 4561095) and transferred to nitrocellulose or methanol activated PVDF membranes. Membranes were blocked in 5% BSA TBS-T or 5% skim milk TBS-T and incubated overnight at 4 °C with the following primary antibodies diluted in 5% BSA or 5% skim milk dissolved in TBS-T: pSer293-PDH (1:1000) (Merck Millipore, Cat. no. ABS204), PDH (1:1000) (Santa Cruz, Cat. no. sc-377092), IDE (1:900) (Abcam, Cat. no. ab32216), LC3 (1:500) (MBL, Cat. no. PM036), pSer403-p62/SQSTM1 (1:500) (Merck Millipore, Cat. no. MABC186-I), p62/SQSTM1 (1:1000) (Cell Signaling, Cat. no. 5114), Synaptophysin (1:500) (Merck Millipore, Cat. no. MAB5258), PSD95 (1:1000) (Cell Signaling, Cat. no. 3409), ULK1 (1:500) (Cell signaling, Cat. no. 8054), pSer555-ULK1 (1:500) (Cell Signaling, Cat. no. 5869), pSer757-ULK1 (1:500) (Cell Signaling, Cat. no. 14202), β3 tubulin (1:2000) (Santa Cruz, Cat. no. sc-80016). After washing with TBS-T for three times, membranes were incubated with fluorescent secondary antibodies (1:10,000–20,000) at room temperature for 1 h. Blots were developed using Odyssey CLx (LI-COR) and the resulting band intensities were quantified by using Image Studio Lite (LI-COR). *n* = 4/genotype.

### Immunofluorescence staining

Brains were dissected from WT and *App*^*NL-G-F*^ mice (*n* = 4/genotype) after perfusion with PBS under anesthetization by isoflurane, and fixed in 10% formalin solution (Merck Millipore, Cat. no: HT501128). Brains were dehydrated and the paraffin-embedded brains were sliced into 4 μm sections by a microtome (Microm HM 360). For triple staining, after deparaffinization and antigen retrieval, sections were stained using Opal 4-Color IHC kit (Akoya, NEL810001KT) according to manufacturer’s instructions with the following primary antibodies: LC3A and B (1:2000) (Novus, Cat. no. NB100-2220), synaptophysin (1:400) (Synaptic Systems, Cat. no. 101 002). After LC3 and synaptophysin staining, sections were incubated overnight at 4 °C with anti-Aβ antibody 82E1 (1:1000) (IBL, Cat. no. 10323) diluted in PerkinElmer antibody diluent/blocking buffer, followed by incubation with anti-mouse IgG antibody conjugated with Alexa Fluor 647 (1:200) (Thermo Fisher Scientific, Cat. no. A-21235) diluted in PerkinElmer antibody diluent/blocking buffer at room temperature for 1 h. For double staining, after deparaffinization and antigen retrieval, sections were blocked with 5% normal goat serum in PBS-T. Sections were first incubated overnight at 4 °C with the primary antibody against LC3A and B (1:2000) (Novus, Cat. no. NB100-2220) diluted in 5% normal goat serum in PBS-T. After washing, sections were incubated with biotinylated anti-rabbit IgG antibody (1:200) (Vector, Cat. no. BA-1000) diluted in 5% normal goat serum in PBS-T for 1 h at room temperature. The signals were amplified by TSA Fluorescein system (Akoya Biosciences, Cat. no. NEL701A001KT) according to the manufacturer’s instructions. Sections were then incubated overnight at 4 °C with anti-Aβ antibody 82E1 (1:1000) (IBL, Cat. no. 10323) diluted in 5% normal goat serum in PBS-T, followed by incubation with Alexa Fluor 546 conjugated anti-mouse IgG antibody (1:200) (Thermo Fisher Scientific, Cat. no. A-11030) diluted in 5% normal goat serum in PBS-T for 1 h at room temperature. Nuclei were counterstained with Hoechst 33342 (1:1000). The images were acquired using Zeiss LSM980 and Nikon fluorescence microscope (Nikon, eclipse E800).

### Isolation of functional mitochondria

Hippocampi were dissected from mouse brains (*n* = 5–6/genotype and age) and washed once in ice-cold PBS. Mitochondria were isolated using discontinuous Percoll density gradient centrifugation as previously described [22]. Briefly, hippocampi were transferred to an 8 ml Dounce tissue grinder of the Potter-Elvehjem PTFE and homogenized approximately 6 times in ice-cold mitochondria isolation buffer (225 mM mannitol, 75 mM sucrose, 1 mM EGTA, 5 mM HEPES–KOH, pH 7.2) supplemented with 1 mg/ml fatty acid-free BSA. The final homogenate was centrifuged at 1100 *g* at 4 °C for 2 min. The supernatant was collected and mixed with fresh 80% Percoll (GE Healthcare, Cat. no. 17-5445-02) prepared in mitochondrial dilution buffer (1000 mM sucrose, 50 mM HEPES–KOH, 10 mM EGTA, pH 7.0), to obtain a 5% Percoll solution, which was further carefully layered on the top of the fresh 10% Percoll. The mitochondrial fraction was pelleted by centrifugation at 18,500 *g* at 4 °C for 10 min. The pellet was then resuspended in 1 ml of mitochondria washing buffer (250 mM sucrose, 5 mM HEPES–KOH, 0.1 mM EGTA, pH 7.2) and centrifuged at 10,000 *g* at 4 °C for 5 min. Mitochondrial pellet was again resuspended in a small volume of ice cold mitochondria washing buffer to achieve a high concentration of mitochondria that was kept on ice for further analysis for a maximum of 3 h. Protein content of isolated mitochondria was quantified using Pierce™ BCA Protein Assay Kit.

### Oxygen consumption rate (OCR) evaluation

OCR was evaluated using the Seahorse Analyzer (Agilent) (*n* = 5–8/genotype and age). This assay is based on the fluorometric detection of oxygen (O_2_) and H^+^ levels in real time via solid state probes in a sensor cartridge which is further equipped with four reagent delivery chambers for injecting compounds during the assay. 2.5 µg of isolated mitochondria diluted in mitochondrial assay solution (MAS; 70 mM sucrose, 220 mM mannitol, 10 mM K_2_HPO_4_, 5 mM MgCl_2_, 1 mM EGTA, 2 mM HEPES–KOH) supplemented with 0.2% (w/v) fatty acid-free BSA were seeded in poly(ethyleneimine)-coated (1:15,000; Sigma–Aldrich, Cat. no: 03880) XFe96 seahorse plates by centrifugation at 2000 g for 18 min at 4 °C. For respiratory coupling, MAS was further supplemented with 10 mM succinate plus 2 μM rotenone to ensure state II respiration, followed by the injection of 4 mM ADP to induce state III, which was then inhibited by addition of 3.2 μM oligomycin. The addition of 4 μM uncoupler FCCP (state IIIu) reflects the maximal respiratory chain activity as well as the maximal substrate oxidation rate. Finally, 4 μM antimycin A was added to fully block the respiratory chain and the residual OCR. To determine electron flow activity, i.e., sequential determination of complexes I-IV-dependent respiration, MAS was supplemented with 10 mM pyruvate, 2 mM malate and 4 µM FCCP. Sequential injection of rotenone (2 μM; complex I inhibitor), succinate (10 mM; complex II substrate), antimycin A (4 μM; complex III inhibitor) and ascorbate/TMPD (N,N,N′,N′-tetramethyl p-phenylenediamine) (10 mM/100 μM; electron donors to cytochrome C/complex IV) were performed to evaluate individual mitochondrial complexes activity [22, 23]. Data was normalized to residual non-mitochondrial respiration.

### Mitochondrial ATP levels

ATP levels were quantified by luminescence using the CellTiter-Glo® Luminescent Assay (Promega, Cat. no. G7571) (*n* = 5–6/genotype and age). This assay relies on the properties of a thermostable luciferase that in presence of ATP mono-oxygenates luciferin and generates a stable glow-type luminescent signal. 10 µg of isolated mitochondria were diluted in PBS to reach 100 µL of final volume. An ATP standard curve was performed using a serial ten-fold dilution of ATP in PBS (1 µM to 10 nM). 100 µL of CellTiter-Glo® Buffer were added to both samples and standard curve and the plate was agitated for 2 min and incubated for 10 min at room temperature before luminescence measurement using the CLARIOstar Plus plate reader (BMG Labtech). Data were analyzed using the MARS Data Analysis Software.

### Calcium retention capacity

Calcium (Ca^2+^) uptake by isolated mitochondria was measured using the Ca^2+^ sensitive probe Calcium Green-5N (*n* = 5–6/genotype and age). Calcium Green-5N is a visible light-excitable Ca^2+^ indicator that does not permeate the mitochondrial membrane and, therefore, exhibits increased fluorescence intensity upon extra-mitochondrial Ca^2+^ binding. Briefly, 10 μg of mitochondria were incubated in K^+^-based buffer (125 mM KCl, 0.5 mM MgCl_2_, 3 mM KH_2_PO_4_, 10 mM HEPES, 0.01 mM EGTA, 3 mM succinate, 3 mM glutamate, 0.1 mM ADP-K, pH 7.4) supplemented with 1 μM oligomycin and 150 nM Calcium Green-5N (Thermo Fisher Sci., Cat. no: C3737) [22, 24]. Mitochondria were then dispensed in a 96-multiwell plate and fluorescence was measured in the microplate reader Fluostar Galaxy (LabVision) by excitation at 506 nm and emission at 523 nm. After a baseline of 3 min, pulses of 10 μM CaCl_2_ were added to mitochondria in 3 min intervals. A decrease in the external Ca^2+^ concentration indicates mitochondrial Ca^2+^ uptake. Mitochondrial Ca^2+^ retention capacity was calculated by the area under the curve after CaCl_2_ pulses, which indicates the amount of extramitochondrial Ca^2+^ taken up by mitochondria.

### Measurement of hydrogen peroxide (H_2_O_2_) production

Levels of H_2_O_2_ were evaluated using the Amplex Red reagent as previously described by us [22] (*n* = 5–6/genotype and age). This assay is based on the oxidation of 10-acetyl-3,7-dihydroxypenoxazine, which is catalyzed by horseradish peroxidase (HRP) in the presence of H_2_O_2_ to produce a red fluorescent oxidation product, resorufin. 5 µg of isolated mitochondria were resuspended in reaction buffer (100 mM sucrose, 100 mM KCl, 2 mM KH_2_PO_4_, 0.01 mM EGTA, 5 mM HEPES–KOH, pH 7.4) supplemented with 3 mM succinate, 3 mM glutamate, 0.1 mM ADP plus 10 μM Amplex Red and 0.5 units/ mL HRP. Fluorescence intensity was evaluated for 20 min (30 sec interval) in the CLARIOstar Plus plate reader (BMG Labtech) using 571 nm excitation and 585 nm emission (37 °C). 2 μM Antimycin A was used as positive control. H_2_O_2_ production was quantified using the slope of the curves.

### Transmission electron microscopy

Ten- to twelve-month-old *App*^*NL-G-F*^ and 22- to 24-month-old *App*^*NL-F*^ mice were anaesthetized and perfused through intracardial perfusion with 2% glutaraldehyde and 1% formaldehyde in 0.1 M phosphate buffer (*n* = 4/genotype). Hemispheres were cut in a brain slicer matrix and coronal slices collected for sectioning. Leica Ultracut UCT or EM UC7 was used to create ultrathin sections, with uranyl acetate and lead citrate used as contrasting agents. For mitochondria and endoplasmic reticulum (ER) analysis, sections were examined at 100 kV using a Tecnai 12 BioTWIN transmission electron microscope. Images were acquired from the hippocampus *Cornu Ammonis* area 1 (CA1) at a primary magnification of 30,000×. Seven different cells were snapped per brain and 30–40 synapses were analyzed per condition. A synapse was considered when the presence of both synaptic vesicles and post-synaptic density were detected. Synaptic vesicles were counted using the cell counter plugin from ImageJ. ER aspect ratio was quantified by dividing the major axis of the ER profile to the minor axis. For the observation of autophagic vacuole accumulation, images were acquired from the hippocampus CA1 at a primary magnification of 16,500× or 26,500×.

### CSF sampling and proximity extension assay (PEA)

Mice were anesthetized with 1.5% isoflurane and thereafter placed in a stereotactic instrument with 120–130° head to body angle (*n* = 5/genotype). A sagittal incision was made posterior to the occipital crest. The dura mater was exposed by a gentle blunt dissection of subcutaneous tissue and muscles under dissection microscope and cleaned by cotton swabs soaked in PBS to remove any blood contamination. The dura mater was punctured with a 27-gauge needle, avoiding visible blood vessels, and CSF was subsequently collected in a glass capillary tube. The collected CSF was inspected under the microscope and discarded in case of detected blood contamination. Samples were collected in low-affinity polypropylene tubes, snap-frozen in liquid nitrogen and stored at −80 °C until further analysis. Levels of 92 proteins in CSF were analyzed using Target 96 Mouse Exploratory Panel with PEA technology, according to manufacturer’s instructions (Olink Proteomics). This dual recognition technique is based on matched pairs of antibodies labeled with complementary DNA oligonucleotide tags, which creates a unique DNA barcode for each antibody pair that is amplified by qPCR.

### Statistical analysis

Statistical analysis was done by using GraphPad Prism version 9 and data were presented as mean ± SEM. *n* refers to the number of unique individual biological samples. Since all samples included in this report were derived from animals, the number of animals used were decided based on a compromise of fulfilling the 3 R recommendation of reducing the number of animals used and our previous experience of the experimental variation observed in the applied techniques. No power analysis was performed. No blinding nor randomization of samples were performed. Non-parametric Kruskal-Wallis tests followed by Dunn’s multiple comparison test or unpaired *t*-test (two tailed) were performed as indicated in the individual figure legends where p-values are reported. Effect size ± SEM is given in figure legends for statistically significant values. Outliers were detected using ROUT method (Q = 1%).

## Results

### Altered transcriptomes in *App*^*NL-F*^ and *App*^*NL-G-F*^ mouse hippocampus

**Fig. 1:**
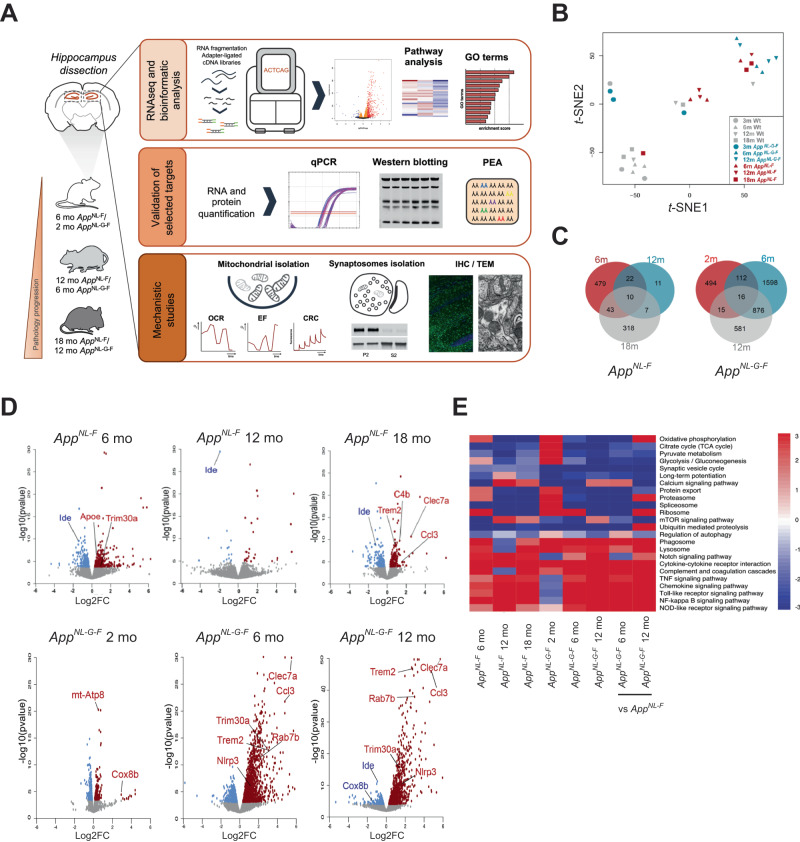
Transcriptome profiling identified Aβ-induced alterations in genes and pathways in hippocampus of *App* knock-in mice. **A** Hippocampi were dissected from two-, six- and 12-month-old *App*^*NL-G-F*^ mice and six-, 12- and 18-month-old *App*^*NL-F*^ mice and age matched WT controls (*n* = 3/genotype). RNA was extracted from dissected hippocampi and cDNA libraries were synthesized. RNA sequencing was performed by a Hiseq 3000 sequencer. Validation studies were performed by RT-qPCR using the same RNA samples used for RNA sequencing. Western blotting with hippocampal brain homogenate and Olink proteomics were conducted for validations at protein level. Mitochondria and crude synaptosomal fraction were isolated for mechanistic studies of mitochondrial and autophagic functions. Mitochondrial dysfunction and autophagic alterations were revealed by electron microscopy. **B** *t-*SNE plot representing difference of *App*^*NL-F*^, *App*^*NL-G-F*^ and WT transcriptomes. Each symbol represents one mouse individual. Each color represents one mouse genotype, red: *App*^*NL-F*^, blue: *App*^*NL-G-F*^, gray: WT, and each shape of symbol represents mouse age, circle: two-month-old, triangle: six-month-old, inverted triangle: 12-month-old, square: 18-month-old. **C** Venn diagram of significantly DEGs in *App*^*NL-F*^ and *App*^*NL-G-F*^ vs WT mice (FDR < 0.1). **D** Volcano plots of the gene expression profiles in different time points of *App*^*NL-F*^ or *App*^*NL-G-F*^ vs WT mice. Red and blue dots indicate significantly up- and downregulated genes (FDR < 0.1), respectively, in *App* knock-in mice. Grey dots indicate non-significantly altered genes. Genes validated by RT-qPCR are highlighted. **E** Heatmap of selected pathways related to AD, glucose metabolism, neuroinflammation and autophagy in *App*^*NL-F*^ vs WT mice (columns 1 to 3), *App*^*NL-G-F*^ vs WT mice (columns 4 to 6) and *App*^*NL-F*^ vs *App*^*NL-G-F*^ mice (columns 7 and 8). The *p*-value of each enriched pathway was converted to a *Z* score. Significantly up- and downregulated pathways have the absolute value of *Z* scores ≥ 1.96.

To gain a comprehensive understanding of how the progression of AD-related pathologies driven by Aβ amyloidosis in the two *App* knock-in mouse models with varying degree of pathology affect gene expression, including genes involved in energy metabolism, we performed a time course mRNA expression profiling of hippocampi of *App*^*NL-F*^ and *App*^*NL-G-F*^ knock-in mice and age matched wild type (WT) controls. This study was designed to enable detection of early alterations prior to plaque deposition in hippocampus (at two and six month-of-age in *App*^*NL-G-F*^ and *App*^*NL-F*^ mice, respectively), pathophysiological changes associated with an established amyloidosis (at six and 12-month-of-age in *App*^*NL-G-F*^ and *App*^*NL-F*^ mice, respectively) as well as changes in the more advanced stages associated with memory impairment (at 12- and 18- month-of-age in *App*^*NL-G-F*^ and *App*^*NL-F*^ mice, respectively) [13, 25] by RNA sequencing (RNA-seq) analyses (Fig. 1A). The RNA-seq results were subsequently confirmed by downstream biochemical, functional, and structural analyses as depicted in Fig. 1A. In total 46,000 transcripts were obtained. We performed *t*-distributed stochastic neighbor embedding (*t*-SNE) which uses high-dimensional data to create a two-dimensional representation of the transcripts. *t*-SNE plot resulted in clustering of transcriptomes of WT mice separately from the transcriptomes of *App* knock-in mice, whereas 18-month-old *App*^*NL-F*^ and six-, 12-month-old *App*^*NL-G-F*^ mice clustered together (Fig. 1B). Notably, the number of differentially expressed genes (DEGs) was much higher in the *App*^*NL-G-F*^ mice, especially at six and 12 month-of-age, as compared to the transcriptome alterations in the *App*^*NL-F*^ mice (Fig. 1C). In *App*^*NL-F*^ mice, the highest number of DEGs were observed at six and 18 months-of-age, prior to and after onset of a robust Aβ plaque pathology, respectively (Fig. 1C). In the *App*^*NL-G-F*^ hippocampus over 600 genes were altered as early as at two-month-of-age, and the number further increased in six- month-old-mice, reaching over 2600 DEGs. Around 1500 DEGs were observed in the 12-month-old mice, though many of the altered genes at this age were already altered at six month-of-age. Volcano plots of transcriptomes from the different cohorts further highlighted the drastic increase in DEGs in *App*^*NL-G-F*^ mice, especially at six and 12 month-of-age (Fig. 1D). Many of the significantly altered genes in the *App* knock-in mice are involved in neuroinflammation (e.g., *Trem2, Ccl3, C4b, Nlrp3*) and lipid and energy metabolism (e.g., *ApoE, mt-Atp8* and *Cox8b*). The transcriptomes were next subjected to Gene Set Enrichment Analysis (Supplemental Fig. 1) which further confirmed significant alterations in pathways associated with energy production including tricarboxylic acid (TCA) cycle and OxPHOS in both *App*^*NL-F*^ and *App*^*NL-G-F*^ mice (Fig. 1E). Other affected pathways included protein homeostasis and synthesis, synaptic vesicles cycle, inflammatory response including cytokine-mediated signaling (Fig. 1E, Supplementary Fig. 1). Interestingly, the pathways evolved differently along disease progression. While inflammatory-related gene expression showed an accelerated upregulation when a robust Aβ pathology is established (Supplementary Fig. 2a), protein processing and synaptic transmission-related pathways displayed a stable downregulation over the course of the disease. We also observed a consistent downregulation of mRNA and protein levels of the Aβ-degrading enzyme insulin-degrading enzyme (IDE) in hippocampi of *App*^*NL-F*^ mice (Supplementary Fig. 2b). On the other hand, energy-related pathways showed an overall upregulation at pre-symptomatic stages, followed by a transcriptional shift to progressive decay, negatively correlating with Aβ extracellular accumulation (Fig. 1E, Supplementary Fig. 1a).

### Early symptomatic *App knock-in* mice display increased hippocampal OxPHOS activity, along with ROS production and calcium handling deficits

**Fig. 2:**
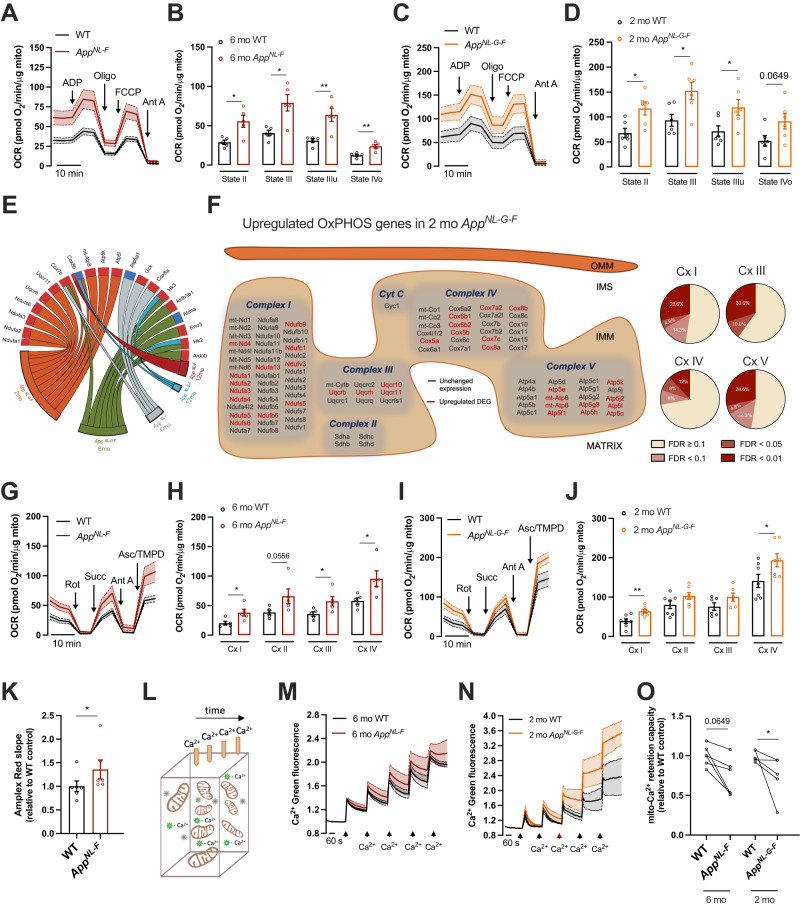
OxPHOS gene expression and activity were upregulated in early symptomatic *App* knock-in mice. **A**–**D** OCR of mitochondria in coupled state, isolated from (**A**, **B**) six-month-old WT and *App*^*NL-F*^ mice and (**C**, **D**) two-month-old WT and *App*^*NL-G-F*^ mice was measured using the Seahorse apparatus, and calculations of state II, state III induced by ADP (4 mM), state IIIu induced by FCCP (4 μM), and state IVo induced by oligomycin (3.2 μM, Oligo) were performed (*n* = 5–6; effect size for *App*^*NL-F*^: state II = 26.58 ± 8.22, state III = 38.49 ± 8.22, state IIIu = 32.79 ± 8.22, state IVo = 11.28 ± 8.22; effect size for *App*^*NL-G-F*^: state II = 49.44 ± 18.87, state III = 58.91 ± 18.87, state IIIu = 48.17 ± 18.87, state IVo = 39.1 ± 18.87). **E** Chord plot of significantly altered genes (FDR < 0.1) included in GO terms related to mitochondrial function. The color of the circle edge boxes indicate up- (red) or down- (blue) regulation. **F** Organization of OxPHOS genes sorted by mitochondrial complex in the mitochondrial cristae. Significantly upregulated genes in two-month-old *App*^*NL-G-F*^ mice are shown in dark red (FDR < 0.1). Percentage of upregulated OxPHOS genes grouped by mitochondrial complex (Cx I, III–V) in two-month-old *App*^*NL-G-F*^ mice based on their FDR value. **G**–**J** Electron flow of uncoupled mitochondria isolated from (**G**, **H**) six-month-old WT and *App*^*NL-F*^ mice and (**I**, **J**) two-month-old WT and *App*^NL-G-F^ mice was evaluated using the Seahorse apparatus. Mitochondrial complex inhibitors and substrates, 2 μM rotenone (Rot), 10 mM succinate (Succ), 4 μM antimycin A (Ant A), and 1 mM ascorbate (Asc)/100 mM TMPD, were sequentially injected to analyze the mitochondrial complex I-IV activities (*n* = 5–8; effect size for *App*^*NL-F*^: Cx I = 17.83 ± 1.19, Cx II = 27.36 ± 1.19, Cx III = 21.58 ± 1.19, Cx IV = 37.64 ± 1.19; effect size for *App*^*NL-G-F*^: Cx I = 24.93 ± 18.87, Cx IV = 52.3 ± 18.87). **K** Levels of H_2_O_2_ in isolated mitochondria from six-month-old WT and *App*^*NL-F*^ mice were fluorometrically quantified using amplex red reagent (*n* = 6; effect size = 0.358 ± 0.231). **L**–**O** Calcium uptake of hippocampal mitochondria was evaluated with the fluorescent probe Calcium-green. Five pulses of 10 μM CaCl_2_ were added to evaluate the mito-Ca^2+^ retention capacity (*n* = 4–5; effect size for *App*^*NL-F*^: 0.278 ± 0.1091; effect size for *App*^*NL-G-F*^: 0.258 ± 0.1253). Statistical significance was analyzed using non-parametric Mann–Whitney test. *p < 0.05, **p < 0.01. OxPHOS oxidative phosphorylation, Cx mitochondrial complex, OCR oxygen consumption rate, IMM inner mitochondrial membrane, IMS inner mitochondrial space, OMM outer mitochondrial membrane.

RNA-seq analysis and subsequent pathway analysis of the hippocampal transcriptomes revealed OxPHOS, which is the final biochemical pathway involved in ATP production, as one of the most upregulated pathways at early symptomatic stages in *App* knock-in mice (FDR = 0.0478 for *App*^*NL-F*^ mice; FDR = 0.0085 for *App*^*NL-G-F*^ mice) (Fig. 1E; Supplementary Fig. S1). Previous multi-omics studies have reported that modules of co-expressed genes related to mitochondrial function positively correlated with Aβ burden and cognitive decline [26]. However, it is still difficult to reconcile how alterations in these large datasets correlate with functional outcomes and pre-clinical pathomechanisms. OxPHOS activity is tightly regulated by a series of events such as post-translational modifications and super-complexes formation [27, 28]. Therefore, it is highly contentious to speculate about mitochondria activity merely based on gene expression profile. We therefore isolated respiring-competent mitochondria by discontinuous Percoll density centrifugation from hippocampus of pre-symptomatic *App*^*NL-F*^ mice and *App*^*NL-G-F*^ mice (six- and two-month-old, respectively) and evaluated the mitochondrial electron flow through electron transport chain (ETC) using the Seahorse XFe96 analyzer, a fluorometric-based assay that detects O_2_ levels in real time (Fig. 2A–D, G–J). Succinate was used as physiological subtract to initiate OxPHOS activity, providing state II respiration. Notably, hippocampus from both *App*^*NL-F*^ and *App*^*NL-G-F*^ mice exhibited increased OCR in state II (*p* = 0.0317 and *p* = 0.026, respectively), indicating elevated mitochondrial respiration under basal conditions. Addition of ADP to fuel complex V further led to an increase in state III (*p* = 0.0317 for *App*^*NL-F*^; *p* = 0.026 for *App*^*NL-G-F*^), suggesting an enhanced capacity to generate ATP (Fig. 2A–D). These data were further confirmed by a positive correlation between ATP levels and state III respiration (*p* < 0.0001) (Supplementary Fig. S3A). Both models also displayed enhanced state IIIu (*p* = 0.0079 for *App*^*NL-F*^; *p* = 0.026 for *App*^*NL-G-F*^) that evaluates the capacity of mitochondria to respire at maximal level after proton gradient uncoupling (Fig. 2B, D). In addition to enhanced OxPHOS-related gene expression, mitochondrial respiration was likely boosted by upstream metabolic pathways such as TCA cycle (FDR = 0.0085 for *App*^*NL-G-F*^ mice) (Fig. 1E). An upregulation of pyruvate dehydrogenase (PDH) activity (detected by a decrease in its inhibitory phosphorylation) in *App*^*NL-G-F*^ hippocampus (*p* = 0.0242) (Supplementary Fig. S3B) further supported this observation, as PDH is a key enzyme that links glycolysis to TCA cycle and which function is directly affected by Aβ [29].

The OxPHOS system is embedded in the inner mitochondrial membrane and is composed of five multiprotein enzyme complexes (I–V) and two electron carriers. A deeper analysis of the OxPHOS system revealed a significant upregulation of both nuclear (e.g.*, Ndufa1-3, Cox7c, Cox8b*) and mitochondrial (e.g.*, mt-Nd4, mt-Atp6, mt-Atp8*) DNA-encoded genes in four out of the five mitochondrial complexes in two-month-old *App*^*NL-G-F*^ mice (Fig. 2E, F, Supplementary Fig. S3C). *App*^*NL-F*^ mice followed the same pattern of upregulation, but the number of metabolism-related DEGs was remarkably lower (Fig. 2E, Supplementary Fig. S3C). Both complex IV and the ATP synthase (complex V) are known to be targeted by Aβ [30, 31]; concordantly, we observed that the most significantly altered genes with FDR < 0.01 belong to complex I, IV and V (Fig. 2F), whereas RT-qPCR analysis showed a tendency of upregulation of some selected genes belonging to mitochondrial complexes I, III–V (Supplementary Fig. S3D). To correlate these data with activities of individual mitochondrial complexes, uncoupled mitochondria were incubated with glycolysis and TCA-derived products, pyruvate and malate, respectively, to drive the activity of mitochondrial complex I. This was followed by the sequential injection of complex II-substrate succinate, complex III inhibitor antimycin A, and complex IV-specific electron donor TMPD combined with ascorbate that ensures TMPD maintains its reduced state (Fig. 2G, I). Despite a significant higher number of DEG in *App*^*NL-G-F*^ mice as compared to *App*^*NL-F*^ mice, both models displayed an increase in activities corresponding to 65 to 88% in complex I (*p* = 0.0159 for *App*^*NL-F*^; *p* = 0.007 for *App*^*NL-G-F*^) and 37 to 65% in complex IV (*p* = 0.0317 for *App*^*NL-F*^; *p* = 0.0156 for *App*^*NL-G-F*^) vs age matched WT mice (Fig. 2H, J). In *App*^*NL-G-F*^ hippocampal mitochondria, complex I and IV activities correlated with RNA-seq data, in which 47.7% and 28% of the genes analyzed from complex I and IV, respectively, were upregulated (Fig. 2F). *App*^*NL-F*^ mice additionally displayed increased activity of complex III (*p* = 0.0317), whereas no changes were detected in complex II for both models (Fig. 2H, J).

Upregulation of ETC increases the risk of electron leakage which may potentiate oxidative stress. Concordantly, Gene Ontology (GO) clustering for biological processes in the two-month-old *App*^*NL-G-F*^ mice revealed upregulation of mitochondrial respiratory complex I assembly, together with changes in oxidation-reduction processes (Supplementary Fig. S3E). To test the functional relevance of this GO terms, we measured mitochondrial hydrogen peroxide (H_2_O_2_) levels, a non-radical reactive oxygen species (ROS), in six-month-old *App*^*NL-F*^ mice, which displayed an increase of both complex I and III activities, the two major sources of mitochondrial ROS. Data revealed a 35% increase in mitochondrial-derived H_2_O_2_ levels in the hippocampus of *App*^*NL-F*^ mice (*p* = 0.026) (Fig. 2K), arguing in favor of enhanced ROS production coupled to OxPHOS activity at this pre-symptomatic stage.

Increased OxPHOS rate associated with ROS synthesis may be indicative of dysfunctional mitochondria despite increased ATP production. To assess mitochondrial optimal function, we performed a mitochondrial Ca^2+^ handling assay. Mitochondria are the second Ca^2+^ storage organelle in the cell after endoplasmic reticulum (ER) and play a vital role to control excitotoxicity under excessive N-methyl-D-aspartate receptors activation [32]. Isolated mitochondria from *App*^*NL-F*^ and *App*^*NL-G-F*^ hippocampi were incubated with the visible light-excitable Ca^2+^ indicator Calcium-Green, and several Ca^2+^ pulses were consecutively performed to test the mitochondrial capacity to store Ca^2+^ (Fig. 2L). Slopes displayed in Fig. 2M, N indicate the rate of Ca^2+^ uptake by mitochondria and cumulative fluorescence intensity in the experimental media which revealed that mitochondria from hippocampus of both *App* knock-in models lose their capacity to accumulate Ca^2+^ much faster than mitochondria from WT mice. Analysis of mitochondrial Ca^2+^ retention capacity confirmed deficient Ca^2+^ handling in mitochondria from hippocampi of *App*^*NL-G-F*^ mice (*p* = 0.0317) and a similar tendency was found in *App*^*NL-F*^ mice (*p* = 0.0649) (Fig. 2O). Overall, these data indicate that mitochondria isolated from hippocampus of both young *App*^*NL-F*^ and *App*^*NL-G-F*^ mice are impaired before the onset of Aβ deposition.

### Increased neuroinflammation in the hippocampus of *App* knock-in mice

Neuroinflammation was previously reported in the *App* knock-in mice [14, 33] and has recently been tightly linked to mitochondrial oxidative metabolism [34]. In agreement, pathway and GO analysis revealed activation of several key inflammatory signaling pathways including TNF, NF-κB, chemokine, Toll-like receptor signaling pathways and complement and coagulation cascade (Fig. 1E, Supplementary Table 1). Analysis using various pathway enrichment tools revealed a dramatic increase in DEGs involved in the neuroinflammatory response in the hippocampus of *App*^*NL-G-F*^ mice from six months-of-age, whereas the inflammatory response was significantly increased in 18-month-old *App*^*NL-F*^ mice, which was further confirmed by RT-qPCR (Supplementary Fig. S4A, B). These data support that the onset of neuroinflammation occurs earlier in *App*^*NL-G-F*^ mice than in *App*^*NL-F*^ mice, most likely due to the more aggressive Aβ pathology induced by the Arctic mutation. Interestingly, no altered inflammation-related GO biological processes were observed at pre-/early symptomatic stages, suggesting that inflammatory cascades are established along with amyloidosis in the *App* knock-in mice (Supplementary Table 1). Inflammatory proteins including cytokines present in the brain can diffuse to CSF and may be utilized as potential markers for brain inflammation. To investigate if inflammatory proteins could be detected and, ultimately, altered in CSF from late-stage *App* knock-in mice, CSF of 18-month-old mice were analyzed by Proximity Extension Assay using a Mouse Exploratory Panel. In agreement with the RNA-seq data showing an increased gene expression of *Ccl3*, Ccl3 protein levels are specifically increased in CSF of *App*^*NL-G-F*^ mice. Other inflammatory markers Ccl5, Ccl20, Tnfrsf12a were altered differently in CSF of the two *App* knock-in mouse models, indicating differences in the inflammatory status in these two models (Supplemental Fig. S4C, Supplementary Table 2).

### Mitochondrial deficits and synaptic structural disorganization characterize late symptomatic *App*^*NL-F*^ and *App*^*NL-G-F*^ mice

**Fig. 3:**
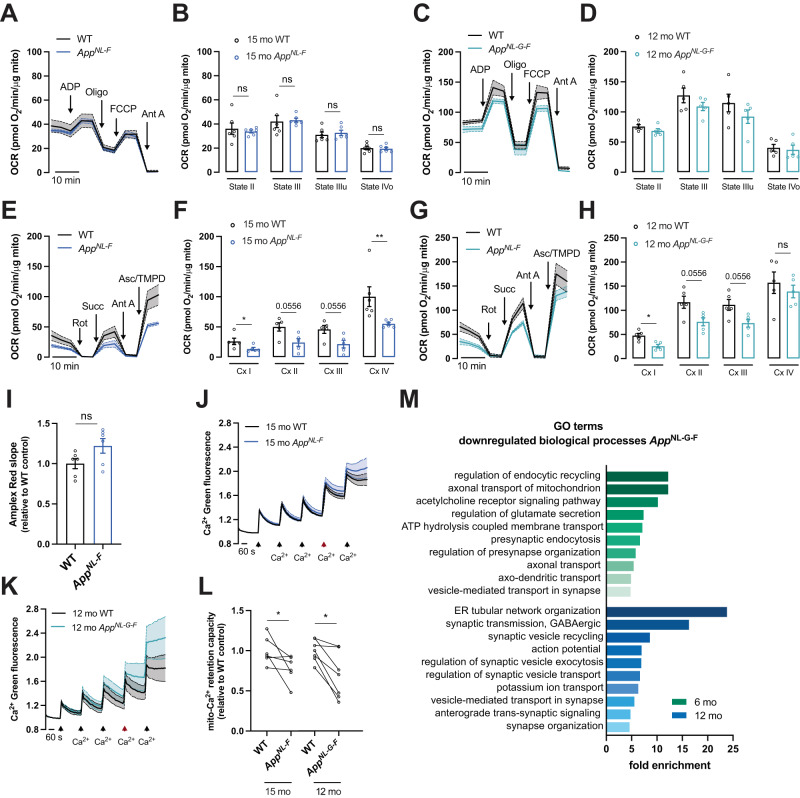
Decay in mitochondrial complexes activities and Ca^2+^ handling capacity characterize symptomatic *App* knock-in mice. **A**–**D** OCR of mitochondria in coupled state, isolated from (**A**, **B**) 15-month-old WT and *App*^*NL-F*^ mice and (**C**, **D**) 12-month-old WT and *App*^*NL-G-F*^ mice was measured using the Seahorse apparatus (*n* = 5–6). **E**–**H** Electron flow of uncoupled mitochondria isolated from (**E**, **F**) 15-month-old WT and *App*^*NL-F*^ mice and (**G**, **H**) 12-month-old WT and *App*^NL-G-F^ mice was evaluated using the Seahorse apparatus (*n* = 5–6; effect size for *App*^*NL-F*^: Cx I = 12.53 ± 12.01, Cx II = 25.86 ± 12.01, Cx III = 24 ± 12.01, Cx IV = 44.9 ± 12.01; effect size for *App*^*NL-G-F*^: Cx I = 22.07 ± 16.71, Cx II = 40.49 ± 16.71, Cx III = 37.89 ± 16.71). **I** Levels of H_2_O_2_ in isolated mitochondria from 15-month-old WT and *App*^*NL-F*^ mice were fluorometrically quantified using amplex red reagent (*n* = 6). **J**–**L** Calcium uptake of hippocampal mitochondria was evaluated with the fluorescent probe Calcium-green. Five pulses of 10 μM CaCl_2_ were added to evaluate the mito-Ca^2+^ retention capacity (*n* = 5–6; effect size: in *App*^*NL--F*^ = 0.224 ± 0.099; in *App*^*NL-G-F*^ = 0.312 ± 0.1195). **M** Significantly downregulated GO biological process terms associated with synaptic and transport functions in *App*^*NL-G-F*^ mice. Statistical significance was analyzed using non-parametric Mann–Whitney test. *p < 0.05, **p < 0.01.

Mitochondrial metabolism in the hippocampus of *App*^*NL-F*^ mice started to decay before 12 months-of-age, whereas in *App*^*NL-G-F*^ mice this effect is observed as early as at six months-of-age and the decline continued upon further aging (FDR = 0.048 at 12 month-of-age and FDR = 0.0105 at 18 month-of-age in *App*^*NL-F*^ mice; FDR = 0.041 at six month-of-age and FDR = 0.006 at 12 month-of-age in *App*^*NL-G-F*^ for OxPHOS) (Fig. 1E). To functionally validate the decay in OxPHOS activity, we evaluated mitochondrial OCR from hippocampus of late symptomatic *App* knock-in and WT mice (Fig. 3A–H). Surprisingly, mitochondrial respiratory states II-IV were preserved in both mouse models (Fig. 3A–D). On the other hand, mitochondrial complex I and IV activities were downregulated by approximately 55% (*p* = 0.0317 in *App*^*NL-F*^*; p* = 0.0159 in *App*^*NL-G-F*^ for Cx I; *p* = 0.0022 for Cx IV in *App*^*NL-F*^), whereas both complexes II and III showed a tendency towards decreased activity of about 45-to-65% (*p* = 0.0556) (Fig. 3E–H), corroborating the RNA-seq data. Despite downregulated OxPHOS, no changes in ROS production were observed (Fig. 3I). These data suggest that mitochondrial respiration is tightly regulated to sustain ATP production even when the expression of individual complexes is affected. Despite that, decreased ability of mitochondria to retain Ca^2+^ (*p* = 0.0411 in *App*^*NL-F*^; *p* = 0.0379 in *App*^*NL-G-F*^) (Fig. 3J–L) advocate for mitochondrial malfunction in hippocampus of *App*^*NL-F*^ and *App*^*NL-G-F*^mice.

**Fig. 4:**
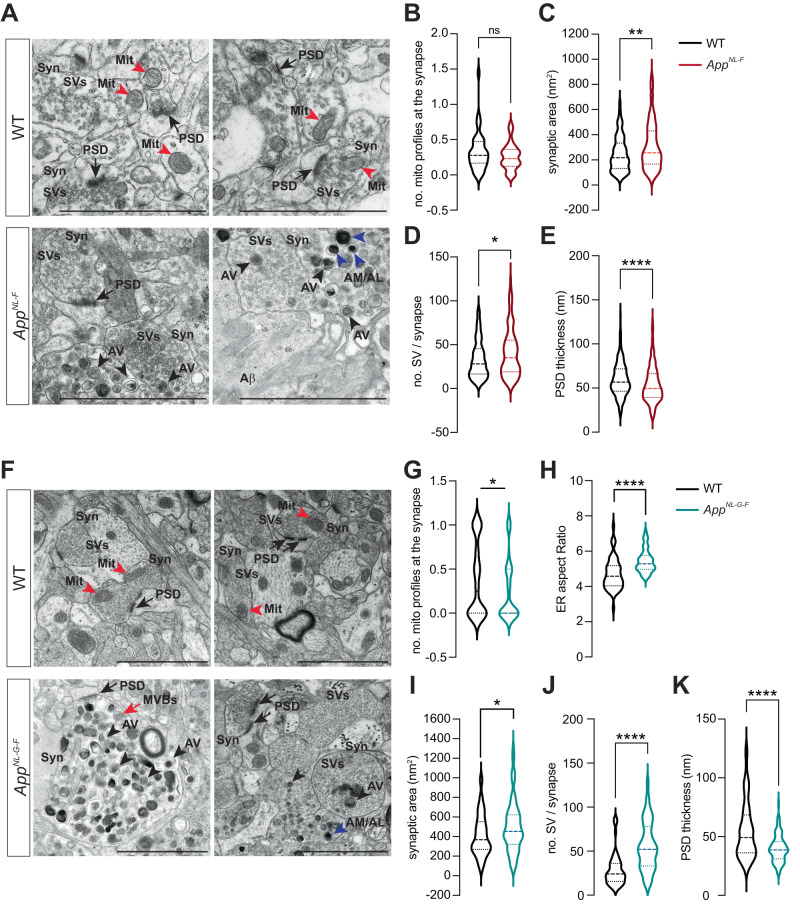
Aberrant synaptic morphology is associated with loss of mitochondria, increase in synaptic vesicles and autophagosome accumulation. **A**, **F** Electron microscopy images of hippocampal CA1 from (**A**) 22-to 24-month-old WT and *App*^*NL-F*^ mice (*n* = 3 including one WT male, with an average of 50 cells and 70 synapses analyzed per genotype; effect size in C = 62.77 ± 19.05; in D = 7.40 ± 2.84; in E = 5.99 ± 1.77), and (**B**) 10- to 12-month-old WT and *App*^*NL-G-F*^ mice (*n* = 4, with an average of 50 cells and 70 synapses analyzed per genotype; effect size in G = 0.16 ± 0.06, H = 0.74 ± 0.157, I = 88.58 ± 43.02; in J = 26.82 ± 4.29; in K = 15.21 ± 2.25). Black arrowhead: AV, blue arrowhead: AM/AL, red arrowhead: Mitochondria, black arrow: postsynaptic density, and red arrow: MVB. Scale bar: 2 µm. Quantification of mitochondrial profiles in the synapses (**B**, **G**), ER aspect ratio (**H**), synaptic area (**C**, **I**), number of synaptic vesicles (**D**, **J**) and post-synaptic thickness (**E**, **K**) in WT and *App* knock-in mice. Statistical significance was analyzed using non-parametric Mann–Whitney test. *p < 0.05, **p < 0.01, ****p < 0.0001. AV autophagic vacuole, AM/AL amphisome/autolysosome, Mit Mitochondria, MVBs multivesicular bodies, PSD postsynaptic density, SVs synaptic vesicles, Syn synapse.

Data from our lab using primary neurons derived from *App*^*NL-F*^ mice indicate that deficits in mitochondrial anterograde movement towards synaptic terminals, precede the decay in mitochondrial respiration, likely contributing to pre- and post-synaptic dysfunction [10]. In agreement, axonal mitochondrial transport and ATP hydrolysis were among the ten most downregulated processes (FDR = 7.78e^-04^ and 1.40e^-04^, respectively), as identified by GO analysis, in the hippocampus of six-month-old *App*^*NL-G-F*^ mice, confirming that a shift in energy metabolism occurs between two to six months-of-age (Fig. 3M, Supplemental Table 1). Moreover, GO analysis of the transcriptomes of six and 12-month-old *App*^*NL-G-F*^ mice also revealed deficits in synapse organization (FDR = 7.66e^-10^ at 6 mo; FDR = 9.25e^-07^ at 12 mo), associated with downregulated synaptic vesicles transport and exocytosis (Fig. 3M, Supplementary Table 1). Synaptic vesicle release is tightly regulated by ATP and Ca^2+^ levels and, therefore, decreased synaptic mitochondria would affect overall synaptic function. Therefore, electron micrographs of hippocampus from 22- to 24-month-old *App*^*NL-F*^ and 10- to 12-month-old *App*^*NL-G-F*^ mice were used to analyze the general organization of the synapse (Fig. 4A, F, Supplementary Fig. S5A, C). No significant alterations were observed in the number of mitochondria profiles at the presynapse in *App*^*NL-*F^ hippocampus (Fig. 4A, B); contrarily, *App*^*NL-G-*F^ hippocampus exhibited a decrease of 44% in the mean average of mitochondrial number at presynaptic terminals (*p* = 0.0227) (Fig. 4F, G). Analysis of the ER network also revealed an increase in the ER aspect ratio, a measure of elongation, together with decreased thickness in *App*^*NL-G-F*^ CA hippocampus (*p* < 0.0001) (Fig. 4H, Supplementary Fig. S5E), in agreement with the GO analysis indicating alterations in ER tubular network organization at this age (Fig. 3M). The extended ER structure can partially explain why the *App* knock-in mice display increased mitochondria-ER contacts sites (MERCS) per mitochondria and consequent mitochondrial fragmentation as previously reported [35]. Furthermore, EM analysis illustrated abnormally enlarged pre-synaptic areas in both *App*^*NL-F*^ (*p* = 0.007) and *App*^*NL-G-F*^ hippocampi (*p* = 0.0366) (Fig. 4C, I), increased number of synaptic vesicles (*p* = 0.0385 in *App*^*NL-F*^; *p* < 0.0001 in *App*^*NL-G-F*^) (Fig. 4D, J), and decreased post-synaptic density thickness (*p* < 0.0001) (Fig. 4E, K). Normalizing the number of synaptic vesicles to synaptic area, revealed that *App*^*NL-G-F*^ mice still exhibits a significantly higher number of vesicles per area, but not *App*^*NL-F*^ mice, emphasizing a stronger pathology in *App*^*NL-G-F*^ mice (Supplementary Fig. S5B, D). Interestingly, around Aβ plaques, the increase in presynaptic area and the number of synaptic vesicles is further pronounced. This area, including both dystrophic neurites and the presynaptic area, also exhibits an accumulation of double membrane autophagic vacuoles (AVs) identified as both autophagosomes and amphisomes/autolysosomes (Fig. 4A, F, Supplementary Fig. S5A, C).

### Autophagy impairment in synapses around Aβ plaques in aged *App knock-in* mice

**Fig. 5:**
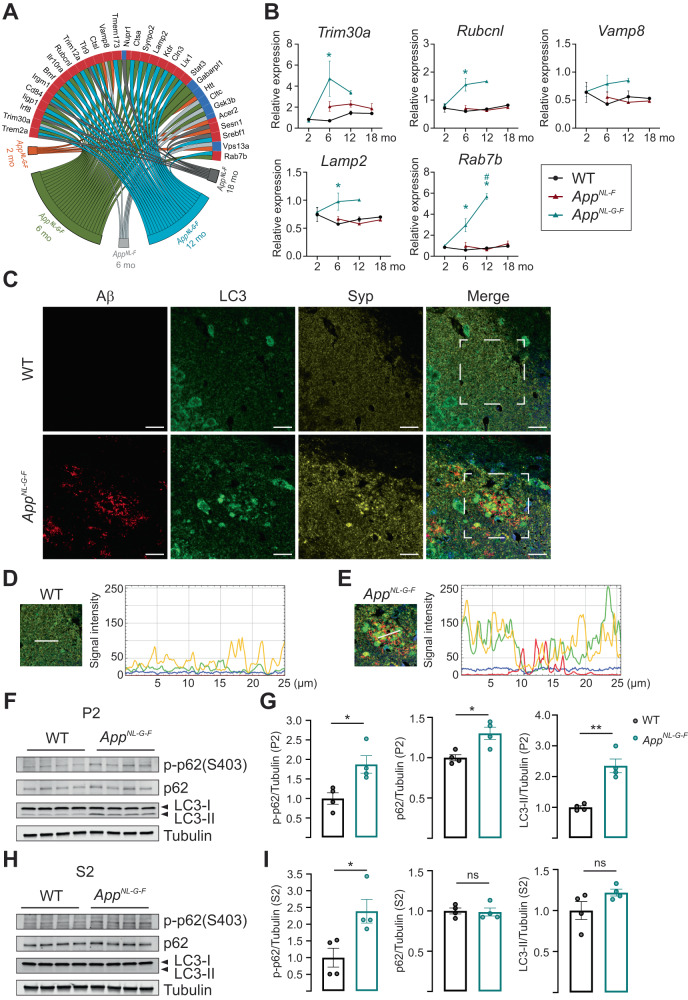
Impaired synaptosomal autophagy in aged *App*^*NL-G-F*^ mice is observed especially around Aβ plaques. **A** Chord plot of significantly (FDR < 0.1) DEGs related to autophagy. The color of the circle edge boxes indicate up- (red) or down- (blue) regulation. **B** The relative mRNA expression of selected genes was normalized to *Tubb3* (*n* = 3; size effect for *Trim30a* 6-month-old *App*^*NL-G-F*^ = 4.004 ± 1.676, for *Rubcnl* 6-month-old *App*^*NL-G-F*^ = 0.9521 ± 0.2321, for *Lamp2* 6-month-old *App*^*NL-G-F*^ = 0.4017 ± 0.1530, for *Rab7b* 6-month-old *App*^*NL-G-F*^ = 2.354 ± 0.6304, 12-month-old *App*^*NL-G-F*^ = 4.910 ± 0.2842, 12-month-old vs 2-month-old *App*^*NL-G-F*^ = 4.678 ± 0.2835). Statistical significance was analyzed using Kruskal-Wallis tests followed by Dunn’s multiple comparison test. *p < 0.05. * vs age matched WT mice, ^#^ vs 2-month-old genotype-matched mice. **C** Co-immunofluorescence staining of Aβ, LC3 and synaptophysin with 12 month-old WT and *App*^*NL-G-F*^ mice (*n* = 4). Red: Aβ, Green: LC3, Yellow: Synaptophysin, Blue: nucleus. Scale bar: 20 µm. **D**, **E** Signal intensity of Aβ (Red), LC3 (Green), Synaptophysin (Yellow) and nucleus (Blue) under the white line of WT and *App*^*NL-G-F*^ mouse brain staining gated in (**C**). **F**–**I** Phospho-p62 (S403), total p62, LC3-I and LC3-II protein levels in hippocampal crude synaptosomal fraction (P2) or soluble fraction (S2) from 12-month-old *App*^*NL-G-F*^ hippocampus were visualized by Western blotting (*n* = 4; size effect for p-p62 in P2 = 0.8713 ± 0.2675 and p-p62 in S2 = 1.387 ± 0.4519, for p62 in P2 = 0.3021 ± 0.08530, for LC3-II in P2 = 1.350 ± 0.2286). Protein levels were normalized to β3-tubulin. Statistical significance was analyzed using unpaired *t*-test. *p < 0.05, **p < 0.01.

The identification of an accumulation of AVs in 22 to 24-month-old *App*^*NL-F*^ and 10 to 12-month-old *App*^*NL-G-F*^ mice prompted us to further investigate the autophagy status in these mice. Macroautophagy, here referred to as autophagy, is an intracellular self-digesting system which is impaired in most neurodegenerative disorders including AD, and directly involved in Aβ metabolism [2, 36, 37]. We therefore assessed the transcriptional alterations of autophagy-related genes in *App* knock-in mice. Indeed, the mTOR signaling pathway, which is a key negative regulator of autophagy and hyperactivated in AD brains [38], was activated both in 12-month-old *App*^*NL-F*^ and *App*^*NL-G-F*^ mice (FDR = 0.0167 for *App*^*NL-F*^; FDR = 0.0121 for *App*^*NL-G-F*^) (Fig. 1E). In addition, the regulation of autophagy pathway was downregulated in 12-month-old *App*^*NL-G-F*^ mice (FDR = 0.0895) (Fig. 1E). We also identified significantly altered autophagy genes already at six months-of-age including *Trim30a, Rubcnl, Lamp2* and *Rab7b* annotated by GO terms and confirmed by RT-qPCR (Fig. 5A, B). Furthermore, the RNA-seq and RT-qPCR data revealed a strong upregulation of the GTPase Rab7b (Fig. 5A, B), which binds to and negatively regulates autophagy through acting on Atg4B thereby controlling LC3-I lipidation, the size of autophagosomes and ultimately autophagy flux [39].

To further analyze the autophagy status and its relation to Aβ plaque pathology and synaptic alterations, we stained by triple immunofluorescence against microtubule-associated protein 1 light chain 3 (MAP1LC3), Aβ and the synaptic marker synaptophysin using brains from 12-month-old WT and *App*^*NL-G-F*^ mice. LC3 exists in the cytosol as a soluble form, LC3-I, and is converted to LC3-II by conjugation with phosphatidylethanolamine upon insertion into the autophagosomal membrane. Hence, LC3-II is used as a maker for phagophores and autophagosomes [40]. Although LC3-I and –II cannot be distinguished by immunofluorescence staining, granular staining of LC3 surrounding the Aβ plaques possibly corresponds to the accumulation of AVs in the dystrophic neurites around the Aβ plaques in the *App*^*NL-G-F*^ mice (Fig. 5C). Interestingly, an accumulation of synaptophysin was also found around the Aβ plaques which partially colocalized with the granular LC3 accumulation (Fig. 5C–E), supporting the EM data showing enlarged presynaptic terminals containing AVs (Fig. 4F, Supplementary Fig. S5C). Next, we evaluated p62/SQSTM1, a receptor for facilitating autophagic degradation of ubiquitinated substrates [40], and LC3 levels in crude synaptosomal fraction which contained mainly presynaptic terminal and postsynaptic membrane as validated by synaptophysin and postsynaptic marker postsynaptic density protein 95 (PSD95). The ratio of synaptophysin to PSD95 was significantly increased in *App*^*NL-G-F*^ mouse brain caused by a reduction of PSD95, as previously reported [41] (Supplementary Fig. S6A–C). Consistent with the triple immunofluorescence staining, LC3-II and p62 were both significantly increased in the hippocampal synaptosomal fraction of *App*^*NL-G-F*^ mice whereas no significant differences of p62 and LC3-II levels were detected in the soluble fraction (Fig. 5F–I). Moreover, p-p62 (S403) levels were also increased in crude synaptosomal fraction of *App*^*NL-G-F*^ mouse brains (Fig. 5F, G). Phosphorylation of p62 at serine 403 (S403) stabilizes the binding between p62 and ubiquitinated protein, therefore enhances degradation of ubiquitinated proteins by autophagy [42]. No autophagy alteration was observed in two-month-old *App*^*NL-G-F*^ mouse brain as determined by p62 and LC3 western blot analysis of synaptosomal fractions and LC3 immunostaining, corroborating the RNA-seq data (Supplementary Figure S6D–H). These data suggest a spatial and time-dependent impairment in autophagy system.

The number of autophagosomes can be increased either by the activation of autophagosome production or by the inhibition of autophagosome degradation. To reveal whether autophagy activation is induced in the synapses in the *App*^*NL-G-F*^ mice, we investigated two phosphorylation sites, S757 and S555 of ULK1, the main regulator of autophagy initiation [43, 44]. Phosphorylation at S757, which inhibits ULK1, was significantly reduced in the synaptosomal fraction of *App*^*NL-G-F*^ mice. However, the level of total ULK1 was also decreased in *App*^*NL-G-F*^ mice and we conclude that no change in synaptosomal autophagy initiation is present in the 12-month-old *App*^*NL-G-F*^ mice (Supplementary Fig. S7A, B). Instead, an inhibition of AVs degradation may explain the accumulation of synaptosomal AVs around the Aβ plaques in aged *App*^*NL-G-F*^ mice which is supported by a decrease in gene expression of several lysosomal vATPases (Supplementary Table 3). Taking all the data together, the synapses of *App* knock-in mice are characterized by an autophagy impairment that is associated with enlargement of synapses containing an increased number of synaptic vesicles but fewer mitochondria which are also dysfunctional.

## Discussion

In this study we aimed to determine the time-wise appearance of AD brain pathologies, with a special focus on pre-symptomatic stages where toxic Aβ aggregates induce dysfunction prior to chronic hippocampal Aβ plaques deposition. This was achieved by analyzing the transcriptome of hippocampus, which is a key region for memory formation, of two *App* knock-in mouse models exhibiting different degrees and progression of AD-like phenotypes including Aβ pathology, neuroinflammation and cognitive impairments. Analysis of the 46,000 obtained transcripts revealed that both *App*^*NL-F*^ and *App*^*NL-G-F*^ mice exhibited substantial alterations in their transcription profiles upon progression of pathology and especially pronounced in *App*^*NL-G-F*^ mice which exhibited more than 2000 DEGs most likely due to the fast formation of toxic Aβ oligomers induced by the Arctic mutation inducing a severe neuroinflammation. Interestingly, an adaptative phase is observed in *App*^*NL-F*^ mice at 12-month-of-age, characterized by lower number of DEGs, which occur between an early compensatory response and chronic Aβ accumulation stages. We hypothesize that this stabilization represents a homeostatic transitory phase that may be due to a shift in Aβ40 aggregation before the insoluble Aβ42 levels reach a plateau [14]. In addition, at 12 months-of-age a shift in Aβ40 aggregation occurs as shown by an increase in Gu-HCL-soluble Aβ40 at the expense of Tris-soluble Aβ. Analysis of the affected pathways revealed that several key cellular functions were significantly affected. These included (1) early changes in mitochondrial function characterized by hypermetabolism, (2) onset of neuroinflammation, (3) decreased mitochondrial function associated with presynaptic-localized autophagy impairment. These changes ultimately lead to an abnormal pre-synaptic organization likely contributing to the synaptic impairment and memory decline in the *App* knock-in mice. By combining transcriptome data with functional analysis, we showed that an early event in the development of the AD-associated pathologies is hypermetabolism mainly characterized by upregulation of OxPHOS. Despite a more severe transcriptomic dysregulation in the *App*^*NL-G-F*^ mouse hippocampus, the functional mitochondrial phenotype was surprisingly even between both knock-in mouse models, suggesting that the distinct Aβ conformation induced by the Artic mutation does not induce additionally enhanced toxicity to mitochondria. OxPHOS alterations have been extensively characterized in postmortem AD brains in the 1990s, with a particular focus on complex IV [45]. Recent machine learning analyses using transcriptomic datasets also predicted AD based on downregulation of specific OxPHOS genes [46]. Despite that, mitochondrial abnormalities are not restricted to AD and have been observed in a variety of other neurodegenerative disorders [47]. A downregulation of nuclear-encoded OxPHOS genes is observed in the hippocampus of AD patients, however MCI subjects show an overall upregulation of OxPHOS genes belonging to complex I, III and IV, similarly to what we have described in our study [48]. Importantly, these changes may be brain-region dependent as studies using frontal cortices observed an increase in mitochondrial-encoded genes belonging to mitochondrial complex III and IV in both MCI and AD patients [49]. This upregulation has been suggested to result from a block of translation, as nuclear-encoded mitochondrial ribosome genes are also downregulated in MCI and AD. However, this hypothesis does not argue in favor of enhanced OxPHOS activity. This hypermetabolic function is likely a feature of pre-symptomatic AD/MCI before clinical conversion to AD associated with an inverse Warburg effect, a compensatory mechanism to maintain energy supply within certain limits under diminished energetic efficiency [50]. Early increase in excitatory and inhibitory presynaptic activities and network hyperactivity in *App* knock-in mouse brain [51–53] may reflect the need for increased ATP supply. Interestingly, we have previously reported enhanced mitochondrial respiration in primary cortical neurons derived from *App*^*NL-F*^ mice [10], suggesting that mitochondrial hyperactivity also occurs in cortex at early stages. Aβ interaction with mitochondria may also be an early event in the pathology progression, inducing this compensatory upregulation. We have previously reported the presence of Aβ42 in mitochondria cristae in the cortex of human patients with mild dementia (Mini-Mental State Examination of 25–27) [54]. During inverse Warburg effect neurons compete for lactate generated by astrocytes, promoting a selective advantage of hypermetabolic neurons and sustaining increased metabolic activity in astrocytes. Concordantly, a large-scale proteomic analysis of AD brains revealed that increased sugar metabolism in astrocytes and microglia emerged as one of the network modules most significantly associated with AD pathology and cognitive impairment [6]. Thus, astrocytes in the *App* knock-in mice may contribute to the early increase in bioenergetics and further studies using specific brain cell populations are required.

We conclude that OxPHOS upregulation is one of the most primary characteristics of neuronal vulnerability leading to manifested oxidative damage [55], which is supported by increased H_2_O_2_ production in pre-symptomatic *App*^*NL-F*^ mice. Mitochondrial-derived ROS is intrinsically linked to neuroinflammation [56] and synaptic dysfunction [57]. These data agree with a previous study describing a negative correlation between glucose metabolism in the hippocampus formation and cognitive performance [58] picturing hypermetabolism as a detrimental maladaptation. Further hypothesis also proposed that a higher basal metabolism may accelerate Aβ deposition [59] however, the lag time between OxPHOS upregulation and Aβ plaque formation in *App*^*NL-F*^ hippocampus contradicts it.

Besides changes in transcription, other underlying mechanisms can influence mitochondrial hyperactivity [60], including increased Ca^2+^ shuttling from ER to mitochondria due to upregulated MERCS [35]. Increased MERCS in both *App*^*NL-F*^ and *App*^*NL-G-F*^ mice hippocampus [35] can account for Ca^2+^ overload susceptibility, also associated with synaptic excitability. Importantly, Ca^2+^ overload has been extensively associated with neuronal death and cognitive decline in different AD models [60, 61]. Therefore, we propose a model where increased OxPHOS associated with unbalanced oxidation and Ca^2+^ buffering dysregulation which, concomitantly with increased neuroinflammatory signals (dependent or independent on mitochondria), eventually lead to energetic decay. Neuroinflammation in *App* knock-in mice have previously been described to comprise both astrocytosis and microgliosis which was further confirmed here by increases in AD-associated *ApoE* and *Trem2* and by activated neuroinflammatory pathways including TNF signaling, toll-like receptor signaling, cytokine signaling and activation of the complement system [14, 33, 62–64]. It is plausible that a feedback cycle exists between ETC activity and inflammatory cascades, as oxidized mitochondrial DNA triggers NLRP3 inflammasome activation, a pathway largely overactivated in hippocampus of symptomatic *App* knock-in mice and other AD models [65] in a mechanism dependent on mitochondrial Ca^2+^ overload [66]. Importantly, females appear to be more vulnerable to the decay in metabolism due to drastic drop in sex hormones at menopause and dysmorphic microglia activation [67, 68]. Further studies using both sexes should address if this phenotype can be reproduced in male *App* knock-in mice.

Axonal vulnerability has been intrinsically linked to neurodegeneration. Our analysis points to a decrease of synaptic mitochondria in *App*^*NL-G-F*^mice, paralleled by enlarged presynaptic areas with a drastically increased number of synaptic vesicles in both *App* knock-in models. This abnormal enlargement of the synaptic vesicle pool could be due to Aβ-induced disruption of vesicle fusion, altered turnover of vesicles or impaired vesicle-mediated transport, as indicated by GO analysis. In addition, decreased mitochondrial-derived local ATP supply could also contribute to the accumulation of synaptic vesicles since the synaptic vesicle cycle is a major consumer of ATP [69]. In fact, the finding of a normal mitochondrial number in the pre-synapse in hippocampus of *App*^*NL-F*^ mice but abnormal in *App*^*NL-G-F*^ mice may explain the milder dysregulation of synaptic vesicle cycle in *App*^*NL-F*^ mice whereas the high oligomerization rate of arctic Aβ lead to higher toxicity in the *App*^*NL-G-F*^ mice. The reduction of post-synaptic density thickness and PSD95 protein level in the synaptosomal fraction of *App*^*NL-G-F*^ mice also corroborates the impairment of synaptic vesicle exocytosis, resulting in a large inactive pool of synaptic vesicles, since PSD morphology is linked to presynaptic terminal function [70]. These substantial morphological alterations in the synapse, which are ATP-dependent dynamic structures, most likely reflect a deficient neuronal transmission leading to memory impairment in the *App* knock-in mice. Indeed, mushroom spine loss has been previously reported in the *App* knock-in mice [71] and electrophysiological characterization of *App*^*NL-G-F*^ mice have shown a desynchronization of kainate-induced gamma oscillation [72]. Interestingly, an accumulation of AVs was observed in the direct vicinity of synapses. This implies a role of autophagy in the maintenance of protein homeostasis in the synapses and its close bearing to mitochondria maintenance through mitophagy may additionally indicate a role for autophagy in quality control of synaptic mitochondria which deteriorates in aged *App*^*NL-G-F*^ mice. This massive accumulation of AVs around Aβ plaques in dystrophic neurites and synapses is in line with an accumulation of AVs in AD brain [37]. Autophagosomes formed in the presynaptic region are transported retrogradely to the cell body for degradation through fusion to lysosomes [73]. Notably, retrograde transport of autophagosomes is disturbed in AD [73] and the downregulation of axonal transport identified by the GO analysis could be a cause of AV accumulation in *App* knock-in mice. Alterations in transport could also be due to the reduced ATP levels, associated with mitochondrial loss and synaptic mitophagy impairment [74], leading to a vicious cycle. Concordantly, an increased number of cristaeless (damaged) mitochondria have been reported in synapses of transgenic 5xFAD mice, suggesting impaired mitochondrial degradation [75]. Additionally, acidification required for the final degradative step in the autophagy-lysosomal pathway is largely controlled by vATPases, which impairment has been linked to FAD-causing PS1 mutations and altered early in the development of Aβ pathology [76]. Thus, further functional studies are required to unambiguously reveal the underlying mechanisms of autophagosome accumulation in *App* knock-in mice. The presynaptic autophagy-impairment potentially contributes to the enlargement of pre-synaptic area and increased number of synaptic vesicles observed in both *App*^*NL-F*^ and *App*^*NL-G-F*^ mice which is further supported by pulse chase experiments in *App* knock-in mice that revealed an impaired protein metabolism of presynaptic proteins [77]. Newly synthesized synaptic vesicle proteins are involved in neurotransmitter secretion and aged synaptic vesicle proteins are found in synaptic vesicles in the inactive reserve pool [78]. Impaired autophagy may influence the balance between the active recycling and the inactive reserve pool, for example through decreased synaptic vesicle degradation, and subsequent synaptic vesicle accumulation. This hypothesis is supported by evidence showing that synaptic vesicle number and synaptic vesicle protein turnover are regulated by autophagy [79].

Taken together, we have deciphered the temporal appearance of some of the AD-associated pathological alterations using the *App* knock-in mouse models which includes a striking early compensatory mitochondrial hyperactivity in both mild and more aggressive AD model mice, i.e., *App*^*NL-F*^ and *App*^*NL-G-F*^ mice, followed by a strong neuroinflammation and autophagic decline leading to faulty synapses. We highlight that although the *App*^*NL-G-F*^ mice show a much faster progression of neuropathology, the *App*^*NL-F*^ mice follow a similar mechanistic phenotype but with slower progression. This study provides an important longitudinal-based platform to guide future research for identification of early potential therapeutic strategies for AD aiming at strengthening the synapse through mitochondrial improvement, damping neuroinflammation and enhancing protein homeostasis via autophagy.

### Supplementary information

Supp Fig 1

Supp Fig 2

Supp Fig 3

Supp Fig 4

Supp Fig 5

Supp Fig 6

Supp Fig 7

Supp Table 1

Supp Table 2

Supp Fig and Table legends

Supplemental Table 3

## Data Availability

The RNA-seq data is deposited to the NCBI Sequence Read Archive (SRA) with submission number SUB13790586.
