# Supplementary material for: Mitochondrial hypermetabolism precedes impaired autophagy and synaptic disorganization in App knock-in Alzheimer mouse models
Source: Mol Psychiatry. 2023 Nov 1;28(9):3966–81. doi: 10.1038/s41380-023-02289-4 (PMC10730401; doi:10.1038/s41380-023-02289-4)
Supplement: Supplementary file 10 — Supp Fig and Table legends [file 41380_2023_2289_MOESM10_ESM.docx]

**Supplemental Figure S1. Transcriptome pathway analysis.** Pathway analysis was performed using Gene Set Enrichment Analysis (GSEA) method based on the KEGG dataset. The *p*-value of each pathway was converted to Z score. Significantly up- and downregulated pathways after enrichment have an absolute value of Z scores ≥ 1.96. The pathways are arranged in hierarchical order. Genes highlighted in red is upregulated and blue is downregulated.

**Supplemental Figure S2.** (A) Immunofluorescence staining of Aβ plaques in *App^NL-F^* and *App^NL-G-F^* mouse brains at different ages. Scale bar: 1,000 µm. (B) Relative mRNA expression of *Ide* gene was normalized to *Tubb3* (n = 3, left; size effect for 12 mo *App^NL-F^* = -1.844±0.1395, for 12 mo = -146.4±8.736, for 12 mo vs 2 mo WT= 1.541±0.1867). Statistical significance was analyzed using Kruskal-Wallis tests followed by Dunn’s multiple comparison test. ^*^*p* < 0.05. * *App^NL-F^* mice vs age matched WT controls, ^#^ vs two-month-old WT mice. IDE protein levels in hippocampal homogenates were quantified by Western blotting and normalized to β3-tubulin (n = 3, middle and right; size effect for 6 mo = -73.55±13.85, for 12 mo = -146.4±8.736, for 18 mo = -110.3±19.41). Statistical significance was analyzed using one-way ANOVA followed by Tukey’s multiple comparisons test. ^***^*p* < 0.001, ^****^*p* < 0.0001. * vs age matched WT controls.

**Supplemental Figure S3. Mitochondrial-related heatmap, GO clustering and gene expression analysis reveal significant changes at two months in *App^NL-G-F^* mice.** (A) State III mitochondrial respiration (ADP-induced OCR) significantly correlates with ATP levels, quantified by luminescence assay. (B) Phospho (p)-pyruvate dehydrogenase (PDH) (S293) and total PDH protein levels in hippocampal extracts were quantified by western blotting (*n* = 3; size effect = 0.2852±0.1). (C) Heatmap showing gene expression converted to Z score from the KEGG pathway dataset ´Oxidative Phosphorylation´. Genes belonging to mitochondrial complexes (I-V) are arranged together. Genes highlighted in red are up- and blue are downregulated. (D) Relative mRNA expression of selected genes belonging to different mitochondrial complexes (Cx). Data was normalized to *Tubb3* (n = 3; size effect for Uqcrb 12 mo = 0.6133±0.1634, for Cox8b 6 mo = 1.8±0.48, for Cox8b 12 mo = 1.743±0.481, for mt-Atp8 6 mo = 0.4588±0.1085). Statistical significance was analyzed using Kruskal-Wallis tests followed by Dunn’s multiple comparison test. ^*^*p* < 0.05 vs age matched WT controls, ^#^ vs two-month-old *App^NL-G-F^* mice. (E) Gene Ontology clustering plot for biological processes. The color of the circles corresponds to log_10_(p-value) changes. Statistical significance was analyzed using unpaired *t-*test. ^*^*p* < 0.05.

**Supplemental Figure S4. Neuroinflammation is a major pathologic phenotype in the middle to late stage of *App* knock-in mice.** (A) Chord plot of significantly altered genes (FDR < 0.1) related to inflammatory response. The colors of the circle edge boxes indicate up- (red) or down- (blue) regulation. (B) Relative mRNA expression levels of selected inflammatory genes were normalized to *Tubb3* (n = 3; size effect for *Trem2* WT vs *App^NL-G-F^* at 6 mo = 2.496 ± 0.4695, at 12 mo = 1.522 ± 0.6684, *App^NL-F^* vs *App^NL-G-F^* at 6 mo = 2.265 ± 0.5636, at 12 mo = 2.246 ± 0.7078*,* WT vs *App^NL-F^* at 18 mo = 1.237 ± 0.3509, 2 mo vs 6 mo *App^NL-G-F^* = 3.280 ± 0.5548, for *Clec7a* WT vs *App^NL-G-F^* at 6 mo = 1.713 ± 0.4211, at 12 mo = 3.380 ± 1.177, *App^NL-F^* vs *App^NL-G-F^* at 6 mo = 1.832 ± 0.4300, at 12 mo = 4.157 ± 1.117, 2 mo vs 12 mo *App^NL-G-F^* = 4.188 ± 1.117, for *C4b* WT vs *App^NL-G-F^* at 6 mo = 2.531 ± 1.047, at 12 mo = 8.882 ± 1.122, *App^NL-F^* vs *App^NL-G-F^* at 12 mo = 9.742 ± 1.252*,* WT vs *App^NL-F^* at 18 mo = 2.369 ± 0.4523, 2 mo vs 12 mo *App^NL-G-F^* = 10.62 ± 1.080, for *Ccl3* WT vs *App^NL-G-F^* at 6 mo = 3.643 ± 1.533, at 12 mo = 8.446 ± 2.295, *App^NL-F^* vs *App^NL-G-F^* at 6 mo = 3.872 ± 1.530, at 12 mo = 8.826 ± 2.312*,* WT vs *App^NL-F^* at 18 mo = 3.043 ± 0.6955, 2 mo vs 12 mo *App^NL-G-F^* = 9.215 ± 2.287, for *Chrna7* WT vs *App^NL-G-F^* at 6 mo = -0.6257 ± 0.1307, at 12 mo = -0.4337 ± 0.2211, *for ApoE* 6 mo *App^NL-G-F^* = 0.5843±0.3190). Statistical significance was analyzed using Kruskal-Wallis test followed by Dunn’s multiple comparison test. * vs age-matched WT mice, ^+^ vs age-matched *App^NL-F^* mice, ^#^ vs genotype-matched two-month-old mice, ^*^*p* < 0.05, ^**^*p* < 0.01, ^***^*p* < 0.001. (C) CSF was withdrawn from cisterna magna through the dura mater of 18-month-old mice and analyzed by proximity extension assay technology using mouse exploratory panel to detect the changes in proteins related to inflammation (n=5; size effect for Ccl3 WT vs *App^NL-G-F^* = 0.6914 ± 0.2324, for Ccl5 *App^NL-F^* vs *App^NL-G-F^* = -0.3758 ± 0.1107, for Ccl20 WT vs *App^NL-G-F^* = -1.196 ± 0.1967, *App^NL-F^* vs *App^NL-G-F^* = -1.051 ± 0.1923, for Tnfrsf12a WT vs *App^NL-F^* = 0.4549 ± 0.08801, *App^NL-F^* vs *App^NL-G-F^* = -0.3838 ± 0.1202. Statistical significance was analyzed using Kruskal-Wallis tests followed by Dunn’s multiple comparison test. ^*^*p* < 0.05.

**Supplemental Figure S5**. **Overview of altered synaptic morphology in App knock-in mice.** Merged electron microscopy images of hippocampal CA1 from (A) 22- to 24-month-old WT and *App^NL-F^* mice (n = 3 including one WT male, with an average of 50 cells and 70 synapses analyzed per genotype), and (C) 12-month-old WT and *App^NL-G-F^* mice (n = 4, with an average of 50 cells and 70 synapses analyzed per genotype; effect size in D = 0.046±0.008 , in E = 0.021±0.003). Scale bar represents 2 µm in merged images, and 1 µm in zoom-in images. (B, D) Quantification of synaptic vesicles number normalized to the area of the pre-synapse. (E) Quantification of ER thickness. Statistical significance was analyzed using non-parametric Mann Whitney test. ^****^*p* < 0.0001. AV: autophagic vesicle, AM/AL: amphisome/autolysosome, Mit: Mitochondria, PSD: postsynaptic density, SVs: synaptic vesicles, Syn: synapse.

**Supplemental Figure S6. Unaltered synaptosomal autophagy in two months old *App^NL-G-F^* mice.** (A, B) Protein levels of synaptophysin and PSD95 in hippocampal crude synaptosomal fraction (P2) or soluble fraction (S2) from 12-month-old WT and *App^NL-G-F^* mice were detected by western blotting (n = 4; size effect for PSD95/Tubulin = -0.1738±0.04197, for Synaptophysin/PSD95 = 0.1741±0.03002). (C) Synaptophysin and PSD95 protein level, and synaptophysin vs PSD95 ratio in P2 fraction. (D) Co-immunofluorescence staining of Aβ and LC3 with two-month-old WT and *App^NL-G-F^* mice (n = 4). Red: Aβ, Green: LC3, Blue: nucleus. Scale bar: 100 µm. (E-H) Total p62, LC3-I and LC3-II protein levels in P2 or S2 fraction from two-month-old WT and *App^NL-G-F^* mice were quantified by western blotting (n = 4). Protein levels were normalized to β3-tubulin. Statistical significance was analyzed using unpaired *t*-test. ^**^*p* < 0.01.

**Supplemental Figure S7.** **No change in synaptosomal autophagy initiation in 12-month-old *App^NL-G-F^* mice.** (A) Phosphorylated ULK1 (S757) protein levels and (B) phosphorylated ULK1 (S555) and total ULK1 protein levels as well as their ratios in hippocampal crude synaptosomal fraction (P2) from 12-month-old WT and *App^NL-G-F^* mice were detected by western blot (n = 4; size effect for p-ULK1 (S757)/Total ULK1 = -0.1275±0.04626, for Total ULK1/Tubulin = -0.1790±0.05846). Statistical significance was analyzed using unpaired *t*-test. ^*^*p* < 0.05, ^**^*p* < 0.01.

**Supplemental Table S1**

Significantly DEGs were applied to Gene ontology **(**GO) enrichment analysis using online software AmiGO website (http://amigo.geneontology.org/amigo), and the significant enrichment GO terms was identified using Fisher’s Exact test with Bonferroni corrected *p*-values ≤ 0.05.

**Supplemental Table S2**

Proximity Extension Assay (PEA) were used to analyze CSF derived from wildtype, *App^NL-F^* and *App^NL-G-F^* mice according to the manufacturer's instructions. The table contains the raw data and limit of detection (LOD) is indicated for each protein and measured samples with values under LOD and marked in red.
