# Supplementary material for: Mitochondrial hypermetabolism precedes impaired autophagy and synaptic disorganization in App knock-in Alzheimer mouse models
Source: Mol Psychiatry. 2023 Nov 1;28(9):3966–81. doi: 10.1038/s41380-023-02289-4 (PMC10730401; doi:10.1038/s41380-023-02289-4)
Supplement: Supplementary file 11 — Supplemental Table 3 [file 41380_2023_2289_MOESM11_ESM.docx]

**Supplemental Table 3: Significantly altered vATPase subunit genes in *App^NL-G-F^* mice (FDR < 0.1)**

| Age (months) | Gene name | Log_2_ Fold Change | *p*-value | FDR |
| --- | --- | --- | --- | --- |
| 6 | *Atp6v0d2* | 4.56 | < 0.001 | 0.01 |
|  | *Atp6v1e1* | -0.44 | < 0.001 | 0.02 |
|  | *Atp6v0c* | -0.36 | < 0.01 | 0.03 |
|  | *Atp6v1b2* | -0.46 | < 0.01 | 0.05 |
|  | *Atp6v1c1* | -0.47 | < 0.01 | 0.07 |
|  | *Atp6v1a* | -0.58 | < 0.01 | 0.08 |
|  | *Atp6ap1l* | 1.32 | < 0.01 | 0.08 |
| 12 | *Atp6v0d2* | 8.00 | < 0.001 | < 0.001 |
|  | *Atp6v1a* | -0.47 | < 0.001 | 0.01 |
|  | *Atp6v1g2* | -0.61 | < 0.01 | 0.06 |
|  | *Atp6v1b2* | -0.50 | < 0.01 | 0.08 |
